# Unenhanced magnetic resonance angiography as an accurate alternative in the preoperative assessment of potential living kidney donors with contraindications to computed tomography angiography and to contrast-enhanced magnetic resonance angiography

**DOI:** 10.1590/0100-3984.2019.0013

**Published:** 2020

**Authors:** Fernanda Garozzo Velloni, Patrícia Prando Cardia, Ulysses dos Santos Torres, Marco Antonio Haddad Pereira, Thiago José Penachim, Larissa Rossini Favaro, Miguel Ramalho, Giuseppe D’Ippolito

**Affiliations:** 1 Escola Paulista de Medicina da Universidade Federal de São Paulo (EPM-Unifesp), São Paulo, SP, Brazil.; 2 Diagnósticos da América SA (DASA), São Paulo, SP, Brazil.; 3 Centro Radiológico Campinas, Campinas, SP, Brazil.; 4 Grupo Fleury, São Paulo, SP, Brazil.; 5 Hospital Garcia de Orta, Lisboa, Portugal.

**Keywords:** Computed tomography angiography, Magnetic resonance angiography, Kidney, Kidney transplantation, Diagnostic techniques, urological, Angiografia por tomografia computadorizada, Angiografia por ressonância magnética, Rim, Transplante de rim, Técnicas de diagnóstico urológico

## Abstract

**Objective:**

To evaluate the accuracy of steady-state free precession (SSFP) unenhanced magnetic resonance angiography (MRA) at 1.5 T for the identification of multiple renal arteries, using computed tomography angiography (CTA) as the reference standard.

**Materials and Methods:**

This was a prospective study involving 39 patients (26 males; mean age, 62.6 years) who underwent CTA and unenhanced MRA to evaluate the proximal and middle segments of the renal arteries. The analysis was performed in two phases: the quality of unenhanced MRA images was classified as diagnostic or nondiagnostic for the presence of multiple renal arteries by two independent readers; two other independent readers then evaluated the images previously classified as being of diagnostic quality. The sensitivity, specificity, and overall accuracy of unenhanced MRA were calculated, CTA being used as the reference standard. The kappa statistic was used in order to calculate interobserver agreement.

**Results:**

The image quality of unenhanced MRA was considered diagnostic in 70-90% of the extrarenal arterial segments. The CTA examination revealed 19 multiple renal arteries (8 on the right and 11 on the left). The accuracy of unenhanced MRA for the identification of multiple renal arteries was greater than 90%, with a sensitivity of 72.7-100% and a specificity of 96.3-100%.

**Conclusion:**

Unenhanced MRA provides high quality imaging of the extrarenal segments of renal arteries. This method may be used as an alternative for the evaluation of the renal arteries, given that it has an accuracy comparable to that of CTA.

## Abstract (pt)

**Objetivo:**

Avaliar, prospectivamente, a acurácia da angiografia por ressonância magnética (angio-RM) sem contraste, com a sequência *steady-state free precession* (SSFP) 1,5 T, para a identificação de artérias renais múltiplas, usando para isso a angiografia por tomografia computadorizada (angio-TC) como padrão de referência.

**Materiais e Métodos:**

Trinta e nove pacientes (26 do sexo masculino; idade média de 62,6 anos) foram submetidos a angio-TC e angio-RM para avaliação dos segmentos proximais e médios das artérias renais. A análise dividiu-se em duas partes: inicialmente, a qualidade das imagens de angio-RM foi classificada como diagnóstica ou não diagnóstica por dois avaliadores independentes; posteriormente, outros dois avaliadores analisaram as imagens consideradas diagnósticas quanto à presença de múltiplas artérias renais. Sensibilidade, especificidade e acurácia da angio-RM foram calculadas utilizando-se a angio-TC como referência. Estatística kappa foi utilizada para cálculo da concordância interobservador.

**Resultados:**

A qualidade das imagens de angio-RM foi considerada diagnóstica em 70-90% dos segmentos arteriais extrarrenais. A angio-TC revelou 19 artérias renais múltiplas (8 direitas e 11 esquerdas). A acurácia da angio-RM para identificação de artérias renais múltiplas foi maior que 90%, com sensibilidade variando de 72,7% a 100% e especificidade variando de 96,3% a 100%.

**Conclusão:**

A angio-RM sem contraste proporciona imagens de alta qualidade dos segmentos extrarrenais das artérias renais. Este método pode ser alternativamente utilizado para avaliação renal, com acurácia comparável à da angio-TC.

## INTRODUCTION

Living donor transplantation has become an important treatment option for end-stage renal disease. Because it provides a significant reduction in pain and morbidity, laparoscopic living donor nephrectomy is now considered the technique of choice. However, due to the limited field of view inherent to this technique, a preoperative imaging workup is essential for surgical planning, in order to evaluate the renal anatomy and identify anomalies, one of which is vascular multiplicity^(1-3)^.

Digital subtraction angiography continues to be the gold standard for imaging the renal arteries, having the advantage of being diagnostic and possibly being therapeutic for stenosis^(4)^. However, it is an invasive method that uses ionizing radiation and iodinated contrast agents, which are potentially nephrotoxic^(3)^. The introduction of multidetector computed tomography (CT), which has higher spatial and temporal resolution, has allowed the acquisition of high-quality images, producing results comparable to those of digital subtraction angiography in the assessment of renal vascular anatomy and disease involving the large, medium, and small renal vessels. Nevertheless, CT angiography (CTA) also involves the use of ionizing radiation and iodinated contrast agents^(5,6)^.

One magnetic resonance imaging (MRI) technique, magnetic resonance angiography (MRA) has gained broad acceptance and has been increasingly used as an alternative to CTA. Recent developments in gradient hardware, pulse sequences, multi-array receiver coils, and parallel imaging techniques, as well as improved sequence performance, have allowed high-quality, comprehensive noninvasive renal vascular studies to be performed without exposing patients to ionizing radiation or iodinated contrast agents. Because MRA enhanced with gadolinium-based contrast agents produces high-quality images, it has become a mainstay^(7-12)^. There are, however, specific conditions related to the use of gadolinium-based contrast agents, such as a high risk of nephrogenic systemic fibrosis in patients with renal disease, concerns about gadolinium deposition in the basal ganglia and globus pallidus after repeated administration of gadolinium chelates^(13)^, a higher cost per examination, and a small but non-negligible risk of adverse reactions^(14)^. Although those concerns may not always be applicable to the population of potential kidney donors (usually healthy individuals), contraindications to the use of contrast agents (such as allergy) may also be a concern. Given all of that, previous techniques of unenhanced MRA have re-emerged and new techniques have been developed^(15)^. One such technique is balanced steady-state free precession (bSSFP), which has been proposed as a means of evaluating renal artery anatomy and stenosis^(3,16,17)^.

There have been only a few studies comparing unenhanced MRA with the more well-established technique of contrast-enhanced CTA^(17,18)^, one of which specifically evaluated the role of unenhanced MRA in identifying multiple renal arteries. However, for a new diagnostic strategy to be definitively incorporated into clinical practice, the preliminary results should be examined, replicated, and confirmed in additional studies.

Vascular multiplicity is an important issue in the management of kidney donors, and its impact has yet to be broadly investigated^(19-21)^. Therefore, the purpose of this study was to evaluate the image quality of unenhanced bSSFP MRA, as well as its accuracy and reproducibility in identifying multiple renal arteries, using CTA as the reference standard.

## MATERIALS AND METHODS

This was a single-center, prospective, analytical, observational, cross-sectional study involving a consecutive sample of patients with a minimum age of 18 years and normal renal function (characterized by an estimated glomerular filtration rate above 60 mL/min/1.73 m^2^). Participants were excluded if they were unsuitable candidates for MRI scanning due to standard contraindications (e.g., a pacemaker or metal implants), phobias, or a history of allergy to iodinated contrast. The final sample included 39 patients (26 males; 13 females) with a mean age of 62.6 years (range, 26-87 years). The study was approved by the local institutional review board. All participating patients gave written informed consent.

To avoid memory, contextual, and observation biases, we selected patients scheduled to undergo CTA for a wide range of indications in standard clinical practice: evaluation of aortic aneurysm; hypertension; potential kidney donor; preoperative planning of liver surgery; suspected chronic mesenteric ischemia; renal asymmetry observed on ultrasound; and focal renal lesions. After the CTA study, all patients underwent a complementary unenhanced MRA examination in a 1.5-T MRI scanner. In most cases, both examinations were performed on the same day. For the cases in which this was not possible (due to patient requests, availability of the MRI scanner, etc.), the maximum interval between the two examinations was 30 days.

### Image technique

**Figure 1:**
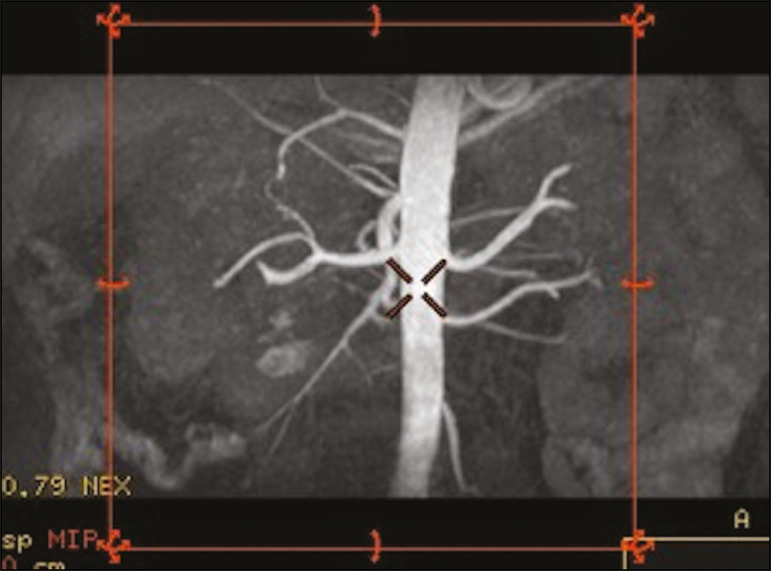
Unenhanced MRA of renal arteries showing the coverage of the bSSFP sequence (focused on the renal artery plane, extending for 12 cm in the craniocaudal direction). MIP, maximum intensity projection.

Unenhanced MRA examinations were performed in a 1.5-T scanner (Signa HDxt; General Electric, Milwaukee, WI, USA) with an 8-channel surface coil and respiratory synchronization. An inflow-sensitive inversion recovery pulse sequence was performed (respiratory-triggered inversion recovery-prepared fat-saturated three-dimensional bSSFP sequence). The sequence was performed during free breathing, and the images were acquired during exhalation (Figure 1), with the following parameters: axial acquisition; field of view, 38 cm; flip angle, 90°; slice thickness, 1.2 mm; echo time, 2.3 ms; repetition time, 4.5 ms; inspiratory time, 1200 ms; reconstruction algorithm, array spatial sensitivity encoding technique; acceleration factor, 2; nominal filling time, 4.36 min; average acquisition time, 5 min (maximum, 8 min); and coverage, 120 mm.

The CTA scans were performed in a 64-slice multidetector scanner (LightSpeed VCT; General Electric), with the following parameters: rotation time, 0.5-0.6 s; pitch, 0.5-0.9; table speed 20.62-39.37 mm/s; slice thickness, 5 mm; interslice gap, 5 mm; reconstruction interval, 0.6 mm; voltage, 120 kV; current, automatic modulation; and noise index, 9.0-10.0 mA. Nonionic iodinated contrast agent was injected intravenously, at a dose of 1.0 mL/kg, by using a dual-head power injector (Stellant; Medrad, Inc., Indianola, PA, USA) at an injection rate of 5.5 mL/s, followed by a 40 mL saline flush, in accordance with a widely adopted protocol described in the literature^(22)^. Automated scan-triggering software (SmartPrep; General Electric) was used in order to start image acquisition. In the CTA examinations, the radiation dose ranged from 2.4 mSv to 13.8 mSv (as a function of abdominal circumference and the use of radiation dose modulation), with a mean of 8.6 mSv.

### Image analysis

All images were reviewed and analyzed on dedicated workstations (Advantage Workstation; General Electric). Initially, all images were displayed with standard images in the axial plane. Multiplanar reformatting was used with or without multiple intensity projection at the discretion of the readers. Image analysis was performed in two phases.

#### Phase 1: evaluation of the unenhanced MRA image quality

Two certified abdominal radiologists with 12 and 9 years of experience, respectively, reviewed the anonymized images. Both were blinded to the patient clinical data and worked independently. The image analysis began with an evaluation of the unenhanced MRA examinations. To reduce the risk of a memory bias, the CTA images were evaluated in random order, after an interval of 21 days, and the readers reached their conclusions regarding the results (adopted as the reference standard) by consensus.

**Figure 2:**
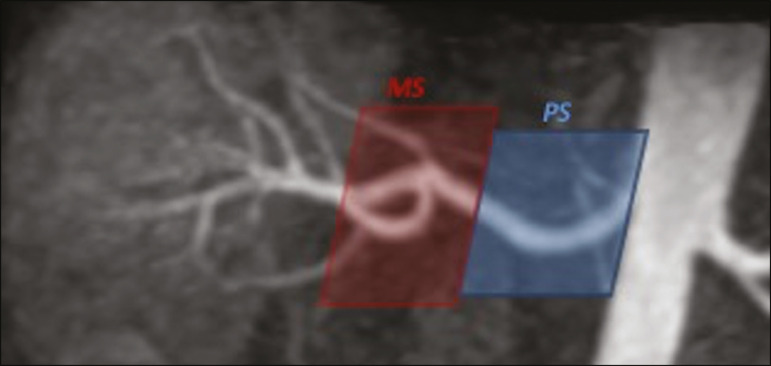
Unenhanced MRA image demonstrating the segmentation of the renal arteries into the proximal segment (PS) and middle segment (MS).

The right and left renal arteries were divided into two segments (Figure 2): the proximal segment (first half) and the middle segment (second half). The quality evaluation was done subjectively and was based on parameters established previously^(23-25)^, including the use of a 4-point scale to classify the degree of vessel wall definition, luminal contrast, and diagnostic confidence: class A, excellent quality (high signal intensity in the arterial lumen-high degree of diagnostic confidence); class B, good quality (moderate signal intensity in the arterial lumen-suitable for diagnosis); class C, moderate quality (minimal signal intensity in the arterial lumen-less suitable for diagnosis); and class D, nondiagnostic quality (no signal in the arterial lumen-insufficient for diagnosis). For statistical analysis purposes, the results were divided into two groups^(26)^: diagnostic (classes A and B) and nondiagnostic (classes C and D).

#### Phase 2: identification of multiple renal arteries

Two other certified abdominal radiologists with 6 and 2 years of experience, respectively, both of whom were blinded to the patient clinical data and previous tests results, performed independent analyses. At this stage of the analysis, only unenhanced MRA images previously classified as being of diagnostic quality (class A or B) were evaluated. The presence or absence of multiple renal arteries was assessed, and the laterality was specified.

### Statistical analysis

Interobserver agreement for qualitative analysis of multiple renal arteries was assessed by calculating the kappa (κ) statistic, which was interpreted as follows^(27)^: κ < 0.4 = poor agreement; κ of 0.41-0.75 = satisfactory agreement; and κ > 0.75 = excellent agreement. The z-test was used in order to determine whether there was a statistically significant difference between the right and left kidneys in terms of the proportion of renal arteries for which the image quality was diagnostic. The level of statistical significance was set at *p* < 0.05. We used CTA as the reference standard to calculate the frequency of multiple renal arteries. The chi-square test was applied to determine the sensitivity, specificity, and overall accuracy of each unenhanced MRA observer independently.

## RESULTS

The study sample comprised 39 patients submitted to CTA and unenhanced MRA. One patient had previously undergone left nephrectomy. Therefore, we evaluated a total of 77 kidneys (39 on the right and 38 on the left).

### Quality of unenhanced MRA images

**Figure 3:**
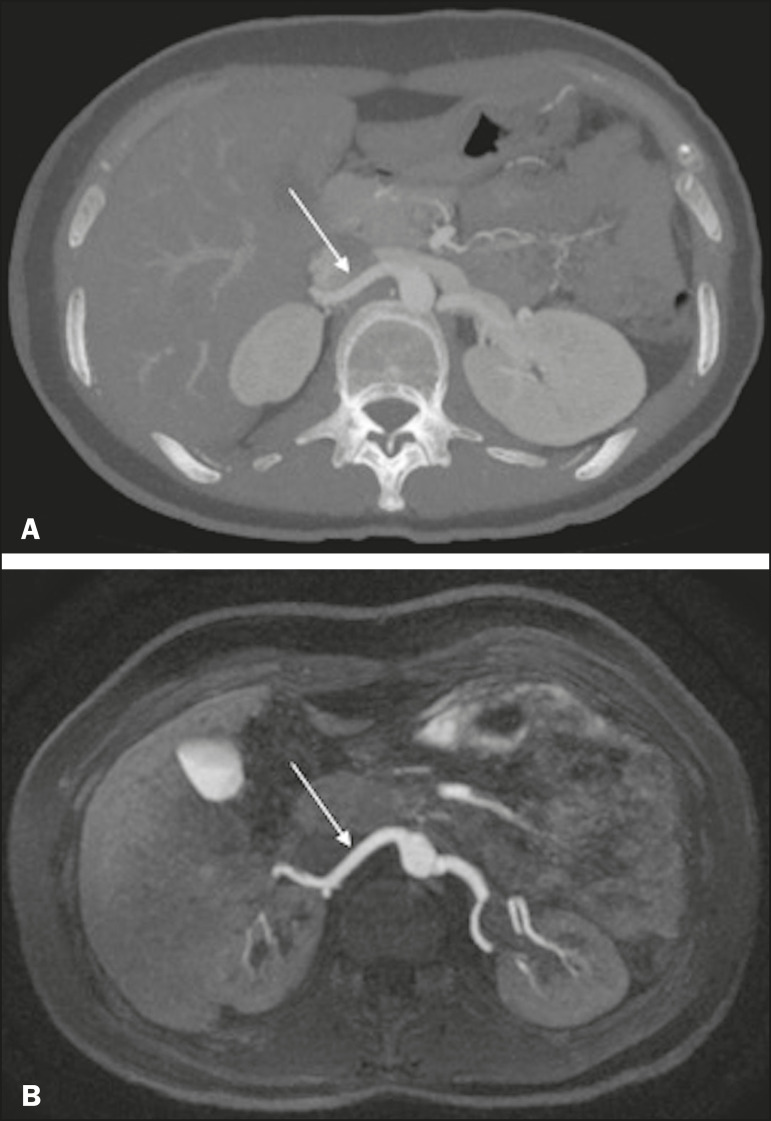
Concordance between the two methods to evaluate the quality of the images. Axial CTA (**A**) and axial unenhanced MRA (**B**) images of the proximalsegment of the right renal artery (arrows) considered to be of diagnostic quality.

Unenhanced MRA showed diagnostic quality (Figure 3) for most of the left and right renal arteries, the proportion of renal arteries for which the image quality was diagnostic ranging from 70% to 90%, depending on the segment analyzed (Table 1). However, there was a statistically significant difference between the diagnostic quality of the middle segments, as well as between the two sides (Table 2).

**Table 1:** Image quality of unenhanced MRA of the segments of the renal arteries, by category.

	Image quality				
	Diagnostic			Nondiagnostic	
Segment	n	(%)		n	(%)
RRA	73	(89.0)		9	(11.0)
LRA	59	(73.8)		21	(26.2)
PRRA	36	(87.8)		5	(12.2)
MRRA	37	(90.2)		4	(9.76)
PLRA	31	(77.5)		9	(22.5)
MLRA	28	(70.0)		12	(30.0)

RRA, (entire) right renal artery; LRA, (entire) left renal artery; PRRA, proximal right renal artery; MRRA, middle right renal artery; PLRA, proximal left renal artery; MLRA, middle left renal artery.

**Table 2:** Comparative analysis of the diagnostic quality of unenhanced MRA images between the right and left renal arteries and their segments.

		Diagnostic					
Pair	Sample	n	(%)	Difference	95% CI	z-value	*P*-value
RRA vs. LRA	RRA	73	(89.0)	0.152	0.0208 to 0.2832	2.3	0.0232
	LRA	59	(73.8)				
PRRA vs. PLRA	PRRA	36	(87.8)	0.103	-0.0772 to 0.2832	1.1	0.2627
	PLRA	31	(77.5)				
MRRA vs. MLRA	MRRA	37	(90.2)	0.202	0.0114 to 0.3926	2.1	0.0378
	MLRA	28	(70.0)				

RRA, (entire) right renal artery; LRA, (entire) left renal artery; PRRA, proximal right renal artery; MRRA, middle right renal artery; PLRA, proximal left renal artery; MLRA, middle left renal artery.

### Identification of multiple renal arteries by unenhanced MRA

On CTA multiple renal arteries were identified in 19 kidneys, 8 on the right and 11 on the left. Table 3 shows the sensitivity, specificity, and overall accuracy of unenhanced MRA, for each of the readers. The overall accuracy of unenhanced MRA was greater than 90%.

**Table 3:** Analysis of unenhanced MRA images regarding the presence of multiple renal arteries.

	Sensitivity		Specificity		Accuracy	
Observer	% (95% CI)		% (95% CI)		%	*P*-value
1 -MRA-R	87.5 (47.3-97.9)		100 (87.1-100)		97.1	< 0.001
-MRA-L	72.7 (39.0-93.6)		100 (82.2-100)		90.0	<0.001
2 -MRA-R	100 (62.9-100)		96.3 (80.9-99.3)		97.1	< 0.001
-MRA-L	81.8 (48.2-97.1)		100 (82.2- 100)		93.3	< 0.001

MRA-R, multiple renal arteries on the right; MRA-L, multiple renal arteries on the left.

**Figure 4:**
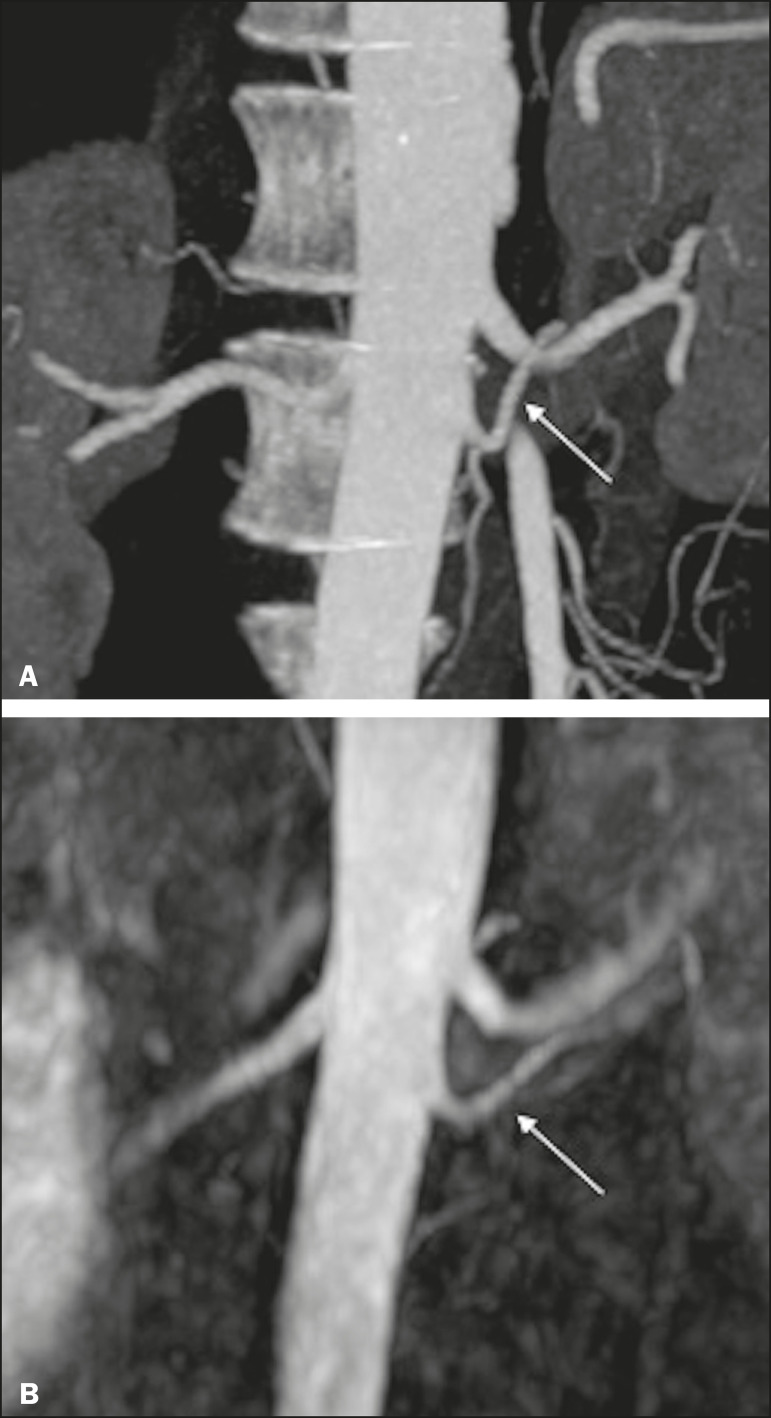
Multiple renal arteries. CTA (**A**) and unenhanced MRA (**B**), showing multiple renal arteries on the left (arrows).

Regarding to the presence of multiple renal arteries (Figure 4), interobserver agreement was excellent, with κ values of 0.83 on the right (95% confidence interval [95% CI]: 0.5-1.0; *p* < 0.001) and 0.75 on the left (95% CI: 0.39-1.0; *p* < 0.001).

## DISCUSSION

Unenhanced MRA is the recommended vascular anatomy imaging method for patients with renal disease, especially those with a glomerular filtration rate < 30 mL/min/1.73 m^2^, who should be examined without the use of exogenous intravenous contrast media^(28,29)^. However, the use of any contrast medium carries potential risks even in patients with normal renal function, and one should consider that when requesting an imaging examination for healthy individuals, such as prospective living kidney donors. In addition, contraindications to the use of contrast agents (such as allergy) can be a concern in this population.

In the present study, unenhanced bSSFP MRA, in comparison with CTA, demonstrated high sensitivity, specificity, and overall accuracy for the identification of multiple arteries, with excellent interobserver agreement, which could contribute to establishing this method as an alternative to CTA for assessing kidney donors.

Although the performance of unenhanced MRA in evaluating renal arteries has been investigated by other authors^(3,14,17,18,30)^, there have, to our knowledge, been only two studies comparing it with CTA, which is the most well-established, robust, and consistent method^(17,18)^. Of those two studies, only one focused on assessing the multiplicity of renal arteries^(18)^. That study involved the use of an extensive, multi-phase, time-consuming MRI protocol, whereas our study was based on a single-phase bSSFP sequence.

As in other studies^(16,22)^, the images of proximal and middle arterial segments obtained by unenhanced MRA in the present study were of diagnostic quality in most cases. It is noteworthy that we found the proportion of nondiagnostic images to be greater for the proximal and middle arterial segments on the left side (22.5% and 30.0%, respectively) than for those on the right side (12.2% and 9.8%, respectively). Those differences may be related to the TI chosen, to differences in blood flow between the left and right renal arteries, or to difficulty in getting an appropriate tagging pulse, which should be positioned in a location that covers both kidneys, which are not typically aligned horizontally^(28)^. These results allow us to state that unenhanced MRA can be an accurate alternative for the identification of multiple renal arteries in individuals with contraindications to CTA or to conventional contrast-enhanced MRA.

Our study has some limitations. Regarding to the technical issues, the exams were performed at 1.5-T equipment. Lanzman et al.^(31)^ compared 1.5-T and 3.0-T unenhanced MRA images of the renal arteries in healthy volunteers. They demonstrated that the quality of the 3.0-T images was significantly better than was that of the 1.5-T images for the third and the fourth branch segments. That may be attributable to the intrinsically higher signal strength, higher signal-to-noise ratio, and longer TI properties of tissues at 3.0-T. However, it should be borne in mind that 1.5-T scanners are much more widely available in clinical practice, as well as that they have been more commonly used in previous studies^(3,17,30)^. Another potential limitation of our study is that it was focused on the extrarenal segments of the renal arteries. Because the internal branches are of smaller caliber, they would represent a greater diagnostic challenge. Nevertheless, it is widely accepted that the most relevant evaluation, in this context, is that of the proximal segments. Finally, despite the lack of a surgical correlation regarding the assessment of multiple arteries, we, like other authors^(14,32)^, believe that using surgical findings as the gold standard may create a bias, since only one of the two kidneys is chosen for harvest, usually the one with less complex anatomy. Therefore, the anatomy of the contralateral kidney would remain unknown, because it would not be submitted to the reference standard method.

In conclusion, most of the unenhanced bSSFP MRA examinations provided images of the extrarenal segments of the renal arteries that were of diagnostic quality, with proper identification of multiple renal arteries in reproducible manner. Our findings support the use of this technique as an alternative for preoperative assessment of the vascular anatomy in a select population of kidney donors, in whom the identification of multiple renal arteries comprises a critical part of the surgical planning.
